# Maternal variants in *NLRP* and other maternal effect proteins are associated with multilocus imprinting disturbance in offspring

**DOI:** 10.1136/jmedgenet-2017-105190

**Published:** 2018-03-24

**Authors:** Matthias Begemann, Faisal I Rezwan, Jasmin Beygo, Louise E Docherty, Julia Kolarova, Christopher Schroeder, Karin Buiting, Kamal Chokkalingam, Franziska Degenhardt, Emma L Wakeling, Stephanie Kleinle, Daniela González Fassrainer, Barbara Oehl-Jaschkowitz, Claire L S Turner, Michal Patalan, Maria Gizewska, Gerhard Binder, Can Thi Bich Ngoc, Vu Chi Dung, Sarju G Mehta, Gareth Baynam, Julian P Hamilton-Shield, Sara Aljareh, Oluwakemi Lokulo-Sodipe, Rachel Horton, Reiner Siebert, Miriam Elbracht, Isabel Karen Temple, Thomas Eggermann, Deborah J G Mackay

**Affiliations:** 1 Institute of Human Genetics, RWTH Aachen University, Aachen, Germany; 2 Faculty of Medicine, University of Southampton, Southampton, UK; 3 Institute of Human Genetics, University Hospital Essen, University Duisburg-Essen, Essen, Germany; 4 MRC Human Genetics Unit, The Institute of Genetics and Molecular Medicine, University of Edinburgh, Edinburgh, UK; 5 Institute of Human Genetics, University of Ulm, Ulm, Germany; 6 Department of Diabetic Medicine, Nottingham University Hospital NHS Trust, Nottingham, UK; 7 Institute of Human Genetics, Bonn, Germany; 8 North West Thames Regional Genetics Service, London North West Healthcare NHS Trust, London, UK; 9 Medical Genetics Center München, München, Germany; 10 Praxis für Humangenetik Homburg, Homburg, Germany; 11 Peninsula Genetics Service, Royal Devon and Exeter Hospital, Exeter, UK; 12 Department of Pediatrics, Endocrinology, Diabetology, Metabolic Diseases and Cardiology, Pomeranian Medical University, Szczecin, Poland; 13 Pediatric Endocrinology, University Children’s Hospital, Tübingen, Germany; 14 Department of Medical Genetics, Metabolism and Endocrinology, The National Children’s Hospital, Hanoi, Vietnam; 15 Department of Clinical Genetics, Cambridge University Hospitals Trust, Cambridge, UK; 16 School of Paediatrics and Child Health, The University of Western Australia, Perth, Western Australia, Australia; 17 Genetic Services of Western Australian and Western Australian Register of Developmental Anomalies, Perth, Western Australia, Australia; 18 School of Clinical Sciences, University of Bristol, Bristol, UK; 19 Wessex Clinical Genetics Service, University Hospital, Southampton, UK

**Keywords:** genomic imprinting, multi-locus imprinting disorder, Beckwith-Wiedemann syndrome, Silver-Russell syndrome, NLRP5, NLRP7, NLRP2, PADI6

## Abstract

**Background:**

Genomic imprinting results from the resistance of germline epigenetic marks to reprogramming in the early embryo for a small number of mammalian genes. Genetic, epigenetic or environmental insults that prevent imprints from evading reprogramming may result in imprinting disorders, which impact growth, development, behaviour and metabolism. We aimed to identify genetic defects causing imprinting disorders by whole-exome sequencing in families with one or more members affected by multilocus imprinting disturbance.

**Methods:**

Whole-exome sequencing was performed in 38 pedigrees where probands had multilocus imprinting disturbance, in five of whom maternal variants in *NLRP5* have previously been found.

**Results:**

We now report 15 further pedigrees in which offspring had disturbance of imprinting, while their mothers had rare, predicted-deleterious variants in maternal effect genes, including *NLRP2*, *NLRP7* and *PADI6*. As well as clinical features of well-recognised imprinting disorders, some offspring had additional features including developmental delay, behavioural problems and discordant monozygotic twinning, while some mothers had reproductive problems including pregnancy loss.

**Conclusion:**

The identification of 20 putative maternal effect variants in 38 families affected by multilocus imprinting disorders adds to the evidence that maternal genetic factors affect oocyte fitness and thus offspring development. Testing for maternal-effect genetic variants should be considered in families affected by atypical imprinting disorders.

## Introduction

Imprinting disorders are caused by genetic and epigenetic variations altering the effective gene dosage of imprinted genes, whose expression is normally restricted by parent of origin.^1^ They include among others the overgrowth disorder Beckwith-Wiedemann syndrome (BWS; overgrowth, macroglossia, exomphalos, hemihypertrophy and predisposition to Wilms tumour), the growth restriction disorders Silver-Russell syndrome (SRS; restricted growth, asymmetry and poor feeding) and Temple syndrome (TS; growth restriction, poor feeding, early puberty and obesity) and transient neonatal diabetes mellitus (TNDM; low birth weight, macroglossia and recurrence of diabetes).

A subset of patients with imprinting disorder show multilocus imprinting disturbance (MLID), that is, DNA methylation disturbance of multiple imprinted genes across the genome, with different imprinting disturbances seen in different patients.^2 3^ Clinically, MLID is generally reported to be associated with a presentation of a ‘classical’ imprinting disorder; for example, ~30% of patients with BWS and hypomethylation of *KCNQ1OT1* TSS DMR (*KCNQ1OT1* transcriptional start site differentially methylated region) are shown to have MLID and ~30% of SRS patients with hypomethylation of *H19* TSS DMR. However, clinically heterogeneous features may affect growth, development, metabolism and behaviour, and some studies have shown an excess of additional clinical anomalies. In most MLID cases, no genetic cause has been found, but some cases are associated with assisted reproductive technology (ART) or with genetic variations in genes expressed during very early embryonic development.^4–8^

During the early embryonic period, there is comprehensive epigenetic reprogramming of sperm and oocyte genomes, zygotic genome activation (ZGA) and onset of differentiation.^9 10^ Before full ZGA (at the eight-cell and two-cell stages in humans and mice, respectively), the early embryo is transcriptionally silent and uses maternally provided transcripts and proteins synthesised abundantly in the growing oocyte during its maturation. Genes of maternal origin with early embryonic expression are said to have ‘maternal effect’, and inactivation of maternal effect genes in mice causes impaired or delayed preimplantation development, frequently leading to embryo demise.

A group of maternally encoded factors, including NLRP5 (Mater), TLE6, OOEP (Moep), KHDC3L (Filia) and PADI6, are among the most highly expressed proteins in both mouse and human oocytes.^11 12^ In mouse, they form a large complex referred to as the subcortical maternal complex (SCMC), which is essential for preimplantation development.^13^ A similar SCMC including KHDC3L, NLRP5, OOEP and TLE6 has been identified in human cleavage-stage embryos.^14^

In humans, maternal effect mutations of NLR family, pyrin domain-containing 5 (*NLRP5*) are associated with heterogeneous outcomes in offspring, including MLID in liveborn children and pregnancy losses and infertility in mothers.^7^ The NLRP protein family includes two other known maternal effect genes: *NLRP7* and *NLRP2. NLRP7* mutations are the major known cause of biparental hydatidiform mole (BiHM), a non-viable reproductive outcome associated with absence of fetal development, abnormal trophoblastic development and complete paternalisation of maternal imprinting,^15^ but *NLRP7* mutations were also described in families with MLID.^6 8^ Maternal mutation of *NLRP2* was found in a family with MLID.^5^ *KHDC3L* maternal mutations are a rare cause of familial BiHM.^16^ Mutations of *PADI6* and *TLE6* were described in mothers undergoing in vitro fertilisation for infertility, whose embryos arrested at the two-cell stage,^17 18^ although imprinting was not analysed in these cases.

Neither human subjects nor murine models are ideal for study of maternal effect genes because, on the one hand, mouse genetics and development do not fully mirror the human situation, and on the other hand, human studies of early development are ethically and technically challenging. Therefore, there is a need to describe human genetic, epigenetic and clinical findings in MLID to inform both clinical diagnosis and murine modelling. Here we present data from an international cohort of families with MLID, where whole-exome sequencing (WES) has identified rare variants in mothers associated with imprinting disorders and other adverse outcomes in offspring.

## Methods

### Study cohort and ethics

Probands were initially referred with a clinical suspicion of an ID and were eligible for research recruitment if initial diagnostic testing of blood-derived DNA revealed methylation disturbance at any imprinted locus. If subsequent testing revealed MLID, further research-based investigation was undertaken, including WES analysis. Thirty-eight such families were included in this study. It should be noted that MLID was not associated with parental unidiploidy (genomewide uniparental disomy) in any case.

Families 1–3, 6 and 11–12 were recruited by the German cooperation partners in the course of the German ‘Imprinting Network’, and the study was approved by the ethical committee at the University Hospital Aachen (EK-302-16). Families 4–5, 7–10 and 13–15 were consented into the study ‘Imprinting disorders – finding out why’ (IDFOW: Southampton and South West Hampshire Research Ethics approval 07/H0502/85) through the UK Comprehensive Local Research network (https://www.southampton.ac.uk/geneticimprinting/informationpatients/imprintingfindingoutwhy.page, accessed October 2017), selected from approximately 1200 individuals recruited from the UK, Europe, Asia, Australia and the USA by virtue of detection of MLID and availability of maternal DNA. Further clinical information on families 5 and 8 was obtained through recruitment into the ‘Study of Adults and Adolescents with Russell-Silver Syndrome in the UK’ (STAARS UK; https://www.southampton.ac.uk/geneticimprinting/informationpatients/staars.page, accessed October 2017).

DNA from a total of 38 families was analysed by WES: 13 from the German consortium and 25 from the UK cohort. In one German and four UK families, maternal *NLRP5* variants were previously reported.^7^ In some family members, insufficient DNA was available for WES; confirmation of variants in these families was performed by Sanger sequencing.

### Exome sequencing

For German pedigrees, Nextera Rapid Capture Exome (FC-140–1083, Illumina, California, USA) was used according to the manufacturer’s protocols. Libraries were sequenced on a NextSeq500 platform with 2×151 paired-end reads and NextSeq high output V.2 chemistry. FASTQ files were generated using the standard Illumina pipeline (V.1.0.0). Paired-end exome sequence reads were aligned to the hg38 human reference genome using Burrows-Wheeler Aligner (BWA-MEM V.0.7.12) to produce binary sequence alignment format (BAM) files, and samblaster (V.0.1.24) was used to remove duplicate reads. Sambamba (V.0.6.6) was applied to sort and index the alignment and Freebayes (V.0.9.21) to determine single-nucleotide variants including SNPs and indel (insertion–deletion) alleles and predict and genotype variants for each sample. Raw variant calls were outputted in variant call format file, and variant filtration was performed for both SNPs and indels to remove low quality and potentially false-positive variants. Variant data were annotated using SnpEff (V.4.2). UK pedigrees were sequenced with the Agilent SureSelect V.5 exome capture kit encompassing 51 Mb of genome sequence (Santa Clara, USA). Paired-end exome sequence reads were aligned to HGRC19 using BWA-MEM (V.0.7.5a), and duplicate reads were removed with Picard (V.1.95). GATK (V.3.0–0)(51) was used to realign and recalibrate BAM files and to predict and genotype variants for each sample. VCF files were annotated using Annovar (V.2013 Aug23) and KggSeq (V.0.6). Sanger sequencing confirmed exome variants and established their inheritance.

### In silico prediction of variant pathogenicity and significance.

The pathogenicity of the variants identified was predicted using the online tools Variant Effect Predictor (http://www.ensembl.org/Tools/VEP) and PROVEAN (http://provean.jcvi.org/genome_submit_2.php?species=human, accessed July 2017), both applied with standard procedures and settings. Pathogenicity predictions are summarised in Results and detailed in online supplementary table 1.

10.1136/jmedgenet-2017-105190.supp1 Supplementary file 1

Hypergeometric analysis was used to estimate the statistical significance of the number of variants found in patients. For each gene in which variants were identified, the number of maternal variants with minor allele frequency (MAF) <0.001 was determined, as well as the cumulative frequency of variants with MAF <0.001 in ExAc-ALL (http://exac.broadinstitute.org, accessed October 2017). The statistical hypergeometric distribution, given as a p value and false discovery rate (FDR) corrected, estimated the likelihood that the number of rare variants (MAF <0.001) identified in patients would be found by chance in the same number of individuals selected at random from ExAc-ALL; for comparison, the hypergeometric p value (FDR corrected) for *NLRP5* was 0.003.

### Epigenetic and epigenomic analysis

Epigenetic analysis was performed by targeted methylation-specific PCR as previously described^19^ or by methylation-specific multiplex ligation probe-dependent amplification assay (ME30, ME032 and ME034; MRC Holland, Amsterdam, The Netherlands). The loci examined included: *DIRAS3* TSS DMR (chr1p31.3); *PLAGL1* TSS alt-DMR (chr6q24); *IGF2R* Int2 DMR (chr6q25); *GRB10* alt-TSS DMR (chr7p12); *MEST* alt-TSS DMR (chr 7q32); *H19* TSS DMR (chr11p15.5); *KCNQ1OT1* TSS DMR (chr11p15.5); *MEG3* TSS DMR (chr14q32); *SNURF* TSS DMR (chr15q11.2); *IGF1R* Int2 DMR (chr15q26); *PEG3* TSS DMR (chr19q13); *GNAS-AS1* TSS DMR (chr20q13.32); and *GNAS* A/B TSS DMR (chr20q13.32).

## Results

Coding variants of *NLRP2* were identified in five mothers, of *NLRP7* in three mothers, of *PADI6* in four mothers and of *OOEP*, *UHRF1* and *ZAR1* in one mother each of offspring with MLID. Despite the overall scarcity of MLID, mothers harboured a statistically significant excess of such variants in *NLRP2*, *NLRP7* and *PADI6*. Table 1 summarises the genetic variants in mothers, the clinical presentations of the offspring and the loci at which imprinting disturbances were detected by targeted testing. Further clinical details are given in online supplementary information, while genetic and epigenetic findings are further detailed in online supplementary tables 1 and 2, respectively. None of the pedigrees reported any family history of imprinting disorders or congenital disorders, and none except family 1 reported consanguinity. Where pregnancies were achieved by ART, this is stated in clinical information and in table 1. All pedigrees are of Caucasian ethnicity, except for families 1 and 13, of Saudi and Southeast Asian origin, respectively.

10.1136/jmedgenet-2017-105190.supp2 Supplementary file 2

**Table 1:** Summary of clinical, genetic and epigenetic features in families with maternal effect variants

Family	Gene	Maternal effect variant*	Hypomethylated loci†	Maternal reproductive history of note	Family history of note	Clinical features of note in proband	Mutation previously reported	Family previously reported: ref (patient)
1	*NLRP2*	NM_017852.4:c. [1479_1480del];[1479_1480del], p.[(Arg493SerfsTer32)];[(Arg493SerfsTer32)] M hom; P1 het; P2 het	*PLAGL1, GRB10, MEST, KCNQ1OT1, GNAS*	Two children affected by MLID, one early abortion (gw 8), two late miscarriages (gw 24 and gw 36), one healthy child	Mother of proband has one healthy sister with three healthy sons	Son: omphalocele, macroglossia, neonatal hypoglycaemia, heart defect, developmental delay. Daughter: macroglossia, dysmorphisms, prominent eyes, developmental delay.	4	
2	*NLRP2*	NM_017852.4:c.[2237del];[=], p.[(Asn746ThrfsTer4)];[=] M, P het	*KCNQ1OT1, H19, MEST*	In vitro Fertilisation, triplet, not monozygotic	NR	SRS (NH-CSS: 6/6)		20 (patient 2) 3 (patient 31)
3	*NLRP2*	NM_017852.4:c.[2860_2861del];[=], p.[(Cys954GlnfsTer18)];[=] M het	*GRB10, MEST, H19, KCNQ1OT1, MEG3,GNAS-AS, GNAS*	NR, only child	Sister with three abortions, no live births	BW at 27 wg 465 g, OFC 32 cm. PNGR, respiratory support for 2 months, gastric tube feeding for first year. Microcephaly, precocious puberty, dysmorphism. Developmental delay. 47,XXY		
4	*NLRP2*	NM_017852.4:c.[314C>T];[=], p.[(Pro105Leu);[=] M het	*PLAGL1, MEST, DIRAS3, IGF1R, IGF2R*	One further child, at least two miscarriages.	Sibling of proband has anxiety disorder	BW 9th centile, neonatal hyperglycaemia, remission at 3 months, childhood height and weight >99th centile, autistic spectrum disorder, speech and language delay		
5	*NLRP2*	NM_017852.4:c.[1885T>C(;)2401G>A], p.[(Ser629Pro)(;)(Ala801Thr)] M, P het both variants	*H19, IGF2R*	One subsequent healthy child, one miscarriage	NR	SRS: NH-CSS 4/6; also bilateral radial anomalies, abnormalities of thumbs, single kidney		19
6	*NLRP7*	NM_001127255.1:c.[2161C>T];[2573T>C]; NP_001120727.1:p.[(Arg721Trp)];[(Ile858Thr)] M het both variants; P not tested	*GRB10, MEST, KCNQ1OT1*	Two early abortions (gw 4 and gw 4) 1 induced abortion (gw 19)	Sister of proband’s mother was also compound heterozygous; one healthy child (born at gw 26), three early abortions (gw 4, gw 6, gw 7); one ongoing pregnancy, ultrasound normal, no MLID, p.(Ile858Thr)	Induced abortion at 19 gw. Omphalocele, shortened humeri. Mesenchymal placenta.	21,22	
7	*NLRP7*	NM_001127255.1:c.[749T>G];[1104T>G]; p.[(Phe250Cys)];[(Ile368Met)] (M compound het; (P Ile368Met het)	*KCNQ1OT1, PLAGL1, IGF2R, MEST, DIRAS3, IGF1R*	NR	NR	BW 91st centile, exomphalos, macroglossia, neonatal diabetes, feeding difficulties in infancy, motor/speech delay, duplex kidneys, hemihypertrophy, scoliosis	20	
8	*NLRP7*	NM_001127255.1:c.[2156C>T];[=], p.[(Ala719Val)];[=] (M het; P het)	*H19, IGF1R, IGF2R*	NR, two additional healthy children	NR	SRS: NH-CSS 5/6	5, 20	
9	*PADI6*	NM_207421.3:c.[902G>A(;)1298C>T], p.[(Arg301Gln)(;)(Pro433Leu)] (M compound het; P not tested)	*H19, MEG3*	NR, only child	Maternal grandpaternal family history of pregnancy loss: one healthy child, one with low birth weight, four stillbirths including a twin pair	BW 2nd centile, preserved OFC, micrognathia, hypotonia and feeding difficulties in infancy. In childhood, facial asymmetry, regrognathia, broad fleshy nasal tip, height 10th–25th centile, weight 90th centile.		19
10	*PADI6*	NM_207421.3:c.[1124T>C];[1639G>A], p.[Leu375Ser)];[(Asp547Asn)] (M compound het; P Asp547Asn het)	*KCNQ1OT1, GRB10, H19, MEST, IGF2R, IGF1R*	NR, only child	NR	BW 90th–97th centile, macrosomia, macroglossia, asymmetry, naevus flammeus, ear creases, developmental delay		3 (patient 31)
11	*PADI6*	NM_207421.3:c.[1046A>G];[=], p.[(Asp349Gly)];[=] (M het)	*H19, IGF2R, GRB10, MEST, MEG3, SNRPN, GNAS-AS, GNAS*	Two healthy children, patient born at term	NR	Referred for testing as SRS, but NH-CSS negative (3/6): no relative macrocephaly, no feeding difficulties, no protruding forehead; developmental delay		
12	*PADI6*	NM_207421.3:c.[433A>G];[=], p.[(Lys145Glu)];[=] (M het)	*H19, IGF2R, MEG3*	NR, only child	NR	SRS: NH-CSS 4/6: no feeding difficulties, no asymmetry		
13	*OOEP*	NM_001080507.2:c.[109C>T];[109C>T], p.[(Arg37Trp)];[(Arg37Trp)] (M hom, P het)	*PLAGL1, IGF2R, DIRAS3, GRB10, SNRPN, IGF1R*	NR	NR	BW <0.4th centile. Hyperglycaemia 1–3.5 months, pelvic renal dilatation, developmental delay		
14	*UHRF1*	NM_013282.4:c.[514G>A];[=], p.[(Val172Met)];[=] (M het, P het)	*H19, PLAGL1, IGF2R, KCNQ1OT1, IGF1R, PEG3, GNAS-AS*	Proband is one of discordant monozygotic twin pair	NR	Discordant monozygotic twin. SRS: NH-CSS 5/6; also kidney failure in infancy, bilateral renal dysplasia		
15	*ZAR1*	NM_175619.2:c.[130G>T];[=], p.[(Glu44Cys)];[=] (M het, P het)	*KCNQ1OT1, GNAS, DIRAS3, IGF1R*	Two healthy siblings, one miscarriage	NR	BW >98th centile, mild macroglossia, consistently high weight (>98th centile)		

47The table summarises clinical, genetic and epigenetic features in families with maternal-effect variants. gw: gestational week; NR: not reported; BW: birth weight; OFC: occipitofrontal circumference; PNGR: postnatal growth restriction; NH-CSS: Netchine-Harbison Clinical Scoring System^47^ (Netchine-Harbison score from 6 parameters: intrauterine growth restriction, postnatal growth restriction, relative macrocephaly, feeding difficulties, asymmetry, protruding forehead). *M: variant detected in mother (by definition); P: variant detected in proband; hom: homozygous; het: heterozygous.

†All loci were tested, but the table lists only loci at which hypomethylation was detected.

### NLRP2

**Figure 1:**
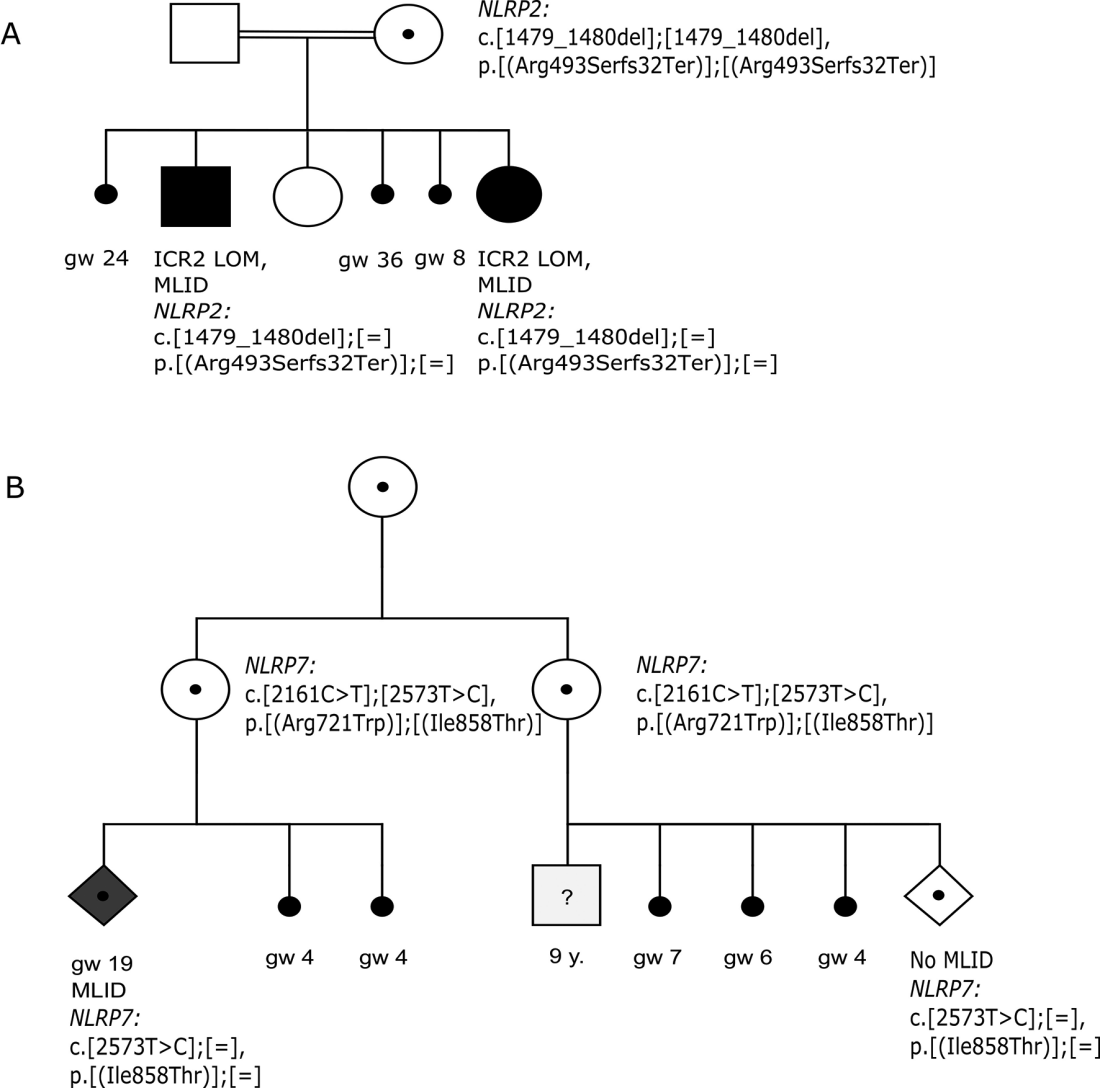
Pedigrees of selected families affected by multilocus imprinting disorders. (A) Family 1 with two BWS-MLID children, who were additionally reported to be developmentally delayed. (B) Family 6 with recurrent pregnancy loss and stillbirths, and one child with MLID and features reminiscent of BWS. BWS, Beckwith-Wiedemann syndrome; gw, gestational weeks; MLID, multilocus imprinting disturbance.

Proband 1 (figure 1A) presented with clinical features of BWS and additionally developmental delay and a heart defect. The mother had a further child with BWS-MLID and also experienced three pregnancy losses. In the mother, the *NLRP2* variant p.(Arg493SerfsTer32) was present homozygously. As expected, the two affected children were heterozygous. This maternal variant was previously reported in a family of Pakistani origin where the offspring were affected by MLID.^5^ Proband 2 with SRS, as previously reported,^20^ is one of trizygous triplets resulting from intracytoplasmic sperm injection. He inherited from his mother the heterozygous frameshift mutation p.(Asn746ThrfsTer4). Proband 3 presented with 47,XXY karyotype, symmetrical growth restriction and developmental delay that were not fully consistent with any specific ID diagnosis. The mother was heterozygous for the stop-gain variant p.(Cys954GlnfsTer18), but the variant was not present in the patient. The sister of the mother experienced three pregnancy losses and had no liveborn children, but no sample was available to determine her carrier status for the variant. Proband 4 affected by TNDM, and developmental delay is the first of three children. One of his siblings has autism; the mother additionally suffered at least two pregnancy losses. The mother but not the proband had the heterozygous *NLRP2* variant p.(Pro105Leu), predicted to be possibly deleterious. Proband 5 with SRS shared with his mother two variants in *NLRP2*: p.(Ser606Pro) and p.(Ala778Thr), each heterozygous; the former was predicted in silico to be deleterious.

### NLRP7

Proband 6 (figure 1B) was a fetus ascertained at 19 weeks of gestation with dysmorphic features suggestive for BWS (omphalocele and placental mesenchymal dysplasia) warranting termination; the mother had two further early pregnancy losses. The mother showed compound heterozygosity for two *NLRP7* variants: p.(Arg721Trp) and p.(Ile858Thr); the variant p.(Arg721Trp) has been reported in families with BiHM and recurrent spontaneous abortion, but the latter’s pathogenicity is unclear.^21 22^ The sister of mother 6 was also compound heterozygous for both variants; she experienced recurrent pregnancy losses but also had a healthy son. Sanger sequencing of the mother of the two sisters showed the presence of the p.(Arg.721Trp) variant only. Proband 7 presented with clinical features of both BWS and TNDM. Her mother had two heterozygous missense variants within the NACHT domain of *NLRP7*: the novel variant p.(Ile368Met) was found only in the mother, but p.(Phe250Cys) was detected in both mother and proband. Of note, missense mutation of p.(Phe250Cys) is reported in BiHM.^23^ Proband 8 had clinical features of SRS. The heterozygous variant *NLRP7*, p.(Ala719Val), identified in the proband and his mother, is predicted as tolerated by in silico tools, but the same variant was identified in a pedigree affected by MLID^6^ and in a mother who suffered four pregnancy losses.^23^

### PADI6

Proband 9 was clinically diagnosed in infancy with SRS, but features of TS emerged in childhood. Her mother had two heterozygous variants in *PADI6*: p.(Arg301Gln) and p.(Pro433Leu), both predicted to be deleterious. No DNA sample from the proband was available to determine inheritance of either variant. Proband 10 showed features of BWS. His mother had two heterozygous variants in *PADI6*: p.(Leu375Ser), not inherited by her son and predicted as deleterious, and p.(Asp547Asn), predicted as benign and inherited by her son. Proband 11 was referred with clinical features reminiscent of SRS. The maternal heterozygous variant p.(Asp349Gly) was identified; this was not inherited by the child. Proband 12 was ascertained with a clinical presentation of SRS; in his mother but not in him, the *PADI6* variant p.(Lys145Glu) was present heterozygously.

### *OOEP*, *UHRF1* and *ZAR1*

Proband 13 had a clinical presentation of TNDM. A missense variant in *OOEP*, p.(Arg37Trp) was present heterozygously in proband 13 and his father and homozygously in his mother. Proband 14, affected by SRS, was one of discordant monozygotic (DMZ) twins; her cotwin was clinically and epigenetically normal. The *UHRF1* variant p.(Val172Met), broadly predicted as deleterious, was present in the mother and both twins. Proband 15 is the third of three children; between the first and second children, the mother suffered one pregnancy loss. The proband had mild macroglossia and high birth weight, but no other features of BWS. She and her mother shared a heterozygous variant in *ZAR1*: p.(Gly44Cys), predicted as possibly deleterious.

## Discussion

We present here 15 families, with imprinting disturbance in offspring and rare maternal variations in proteins expressed abundantly in oocytes and zygotes, and we propose that the maternal variants are associated with the epigenetic and clinical features of the offspring.

Most probands presented features of classic imprinting disorders, prompting epigenetic diagnosis. However, the majority of offspring reported here had additional clinical features, atypical of their primary diagnoses, including developmental delay, autistic features and organ malformations. MLID is inherently heterogeneous, aetiologically, epigenetically and phenotypically.^2^ Further patients and further analysis will be required to determine whether epigenotype:phenotype correlations exist to explain these features, but this is challenging because of the requirement of testing in specific tissues and at key developmental times to confirm the causal relationship. For example, of five probands with hypomethylation of both *H19* TSS DMR and *KCNQ1OT1* TSS DMR, three presented with SRS and one with BWS. The clinical presentation of each patient may have reflected the most severe methylation disturbance in that patient or the pattern of epigenetic disturbances in critical somatic tissues for the two disorders.

One proband (pedigree 14) is a DMZ twin. The incidence of twinning is elevated in imprinting disorders, particularly BWS; the majority are DMZ twins with methylation loss, and MLID is also over-represented in these patients.^24 25^ We previously described a proband with MLID who was a DMZ twin, whose mother had a variant in *NLRP5*.^7^ The addition of this present case supports the hypothesis that MZ twinning is connected with epigenetic disturbance in early development and, in some cases, a genetic predisposition.

Offspring of mothers with maternal effect variants showed heterogeneous and mosaic disturbance of both maternal and paternal genomic imprints. This epigenetic heterogeneity contrasts with the relatively consistent MLID characteristic of recessive *ZFP57* mutation,^4^ or the complete loss of maternal methylation seen in hydatidiform moles,^26^ and suggests that these variants affect not gametic establishment but postzygotic maintenance of imprints. Not all offspring had clinical features of imprinting disorders (although epigenetic disturbance might be present in untested, clinically unaffected siblings), suggesting that the penetrance of the maternal effect was modified by other genetic or environmental factors.

In our cohort, analysis of imprinted DNA methylation was only possible in accessible, somatic tissues. It is possible that maternal effect mutations disturb the whole process of epigenomic reprogamming in the embryo and lead to a ‘crisis’ in its development. If the embryo survives, ongoing differentiation and development overwrite these epigenetic errors, leaving only imprint changes as evidence of the crisis. Indeed, in cases where little or no imprinting disturbance is detectable in accessible tissue, the affected individual may go undiagnosed. Model animal studies are required to determine whether maternal effect mutations impact the embryo epigenome, and how epigenomic disturbance correlates with developmental outcomes. It is also noteworthy that two probands had genomic alterations: one showed 47XXY and one a duplication of chr20. Mouse models deficient in the oocyte protein *Filia* showed increased genomic instability.^27^ While whole-genome analyses were not undertaken in all families, it is possible that the stability as well as the reprogramming of the genome is compromised by maternal effect mutations.

*NLRP7* and *NLRP2* are among several NLR gene family members tandemly located on human chromosome 19. Some NLRPs are involved in humoural immunity^28^; others are expressed abundantly and almost exclusively in the oocyte.

Maternal mutations of *NLRP7* are associated with BiHM.^21–23 29 30^ Women with inactivating *NLRP7* mutations normally have no liveborn children, but pregnancies and live births have been reported in women with missense or splicing mutations, indicating that residual NLRP7 function is compatible with human development.^6 8 20^*NLRP7* has no murine homologue; in the human genome, it is adjacent to *NLRP2* and is likely to represent a recent genomic duplication from it.^31^ Thus, human NLRP2 and NLRP7 proteins may divide, or execute redundantly, the murine function of NLRP2. *Nlrp2* knockdown in mouse germinal vesicles gave rise to embryos that arrested between the two-cell and eight-cell stage.^32^ In a murine *Nlrp2* knockout model, females showed atresia of ovarian follicles, reduced fertilisation rates, abnormal early embryogenesis, delay or failure in blastocyst formation and prenatal or perinatal death with a heterogeneous range of growth and developmental defects, together with methylation disturbance (both hypomethylation and hypermethylation) at imprinted loci.^33^ An independent *Nlrp2*-knockout mouse showed a marked decline in female fertility with age.^34^ Taken together, these observations suggest that functional deficit of Nlrp2 impacts early embryogenesis, leading to a gradation of subviable and nonviable outcomes, associated with altered epigenetic reprogramming, and susceptible to environmental modulation, for example, by maternal age.

Nlrp2 and Nlrp5 are components of the SCMC in mouse, along with Filia (Khdc3l), Moep, Tle6 and Padi6.^13 14 35–44^ In mouse, maternal ablation of SCMC components causes developmental failure of offspring. Ablation of maternal Mater (*Nlrp5*) causes embryonic arrest at the two-cell stage.^35 36^ Maternal-null *Filia* embryos have defective zygote spindle assembly and chromosome alignment, causing delayed mitosis, gross aneuploidy and reduced maternal fertility.^27^ Maternal-null *Moep* (the murine homologue of OOEP) embryos show cell division defects resulting in arrest at the two-cell to four-cell stage.^37^ Tle6 is involved in protein kinase A signalling during oocyte maturation.^38^ Maternal ablation of murine Padi6 leads to disrupted zygotic localisation of ribosomal components, loss of stored mRNA, reduced transcription and translation and developmental arrest at the two-cell to four-cell stage.^39 40^ In our cohort, we found no maternal variants in *TLE6* or *KHDC3L* but four families with rare variants in *PADI6* and one family with a variant in *OOEP*. Of note, the Southeast Asian ethnicity of pedigree 13 raises the possibility that the *OOEP* variant in this family may be under-represented in public databases (dbSNP and ExAC) and unrelated to Proband 13’s clinical presentation.

Additionally, we found variants in two other maternal effect proteins: UHRF1 and ZAR1. Murine Zar1 is expressed in the oocyte and required for progression to the two-cell stage.^41^ Zar1 binds the 3′ of mRNAs and, in vitro, suppresses their translation.^42^ Murine Uhrf1 (Np95) associates with replicating DNA and recruits the DNA maintenance methyltransferase Dnmt1, which preferentially methylates hemimethylated DNA.^43 44^*Nlrp2*-null female mice showed altered localisation of Dnmt1 in oocytes and preimplantation embryos as well as disturbed DNA methylation at imprinted loci.^33^ This observation is consistent with reduced function of UHRF1 impairing maintenance methylation in the early embryo, leading to stochastic and mosaic DNA methylation loss which, at imprinted loci, would not be re-established later in development.

The small number of families and the heterogeneity of the maternal genetic variants and offspring outcomes preclude correlations between genotype, epigenotype and phenotype. This study identified coding variants, including homozygosity and heterozygosity for non-sense and missense mutations. Of six families with maternal homozygous/compound heterozygous mutations (families 1, 6, 7, 9, 10 and 13), three (families 1, 7 and 9) had family histories of non-viable reproductive outcomes as well as children affected by MLID, suggesting that these families have a trend to greater severity of affectedness. In mothers with heterozygous variants, affectedness of offspring may be partly contingent on environmental factors. In pedigrees where no plausible genetic variants were found in mothers or probands, other genetic effects may remain to be identified; otherwise, MLID may be caused by environmental factors such as maternal lifecourse or age, the age of the oocyte preovulation or postovulation,^45 46^ or ART (though in 20 pedigrees in whom variants were not found, ART was reported in only one, and unknown in two others; data not shown).

It will be challenging to prove the functional consequences of these variants, because most of the genes concerned are expressed only in oocyte and early embryo, their expression and function may differ between humans and mice and obvious technical, practical and ethical restrictions limit the analyses that can be performed in humans. However, there is strong justification for reporting these variants and examining the potential relevance of maternal effect variation for female reproductive health and rare disease.

Importantly, current databases of genetic variants are not reliable for considering maternal effect variants, as the individuals carrying these variants are likely to develop normally; it is their offspring or fertility that are affected, and these data are not systematically recorded in any international database to date. Women in this cohort had pregnancy losses as well as children affected by MLID. It is possible that maternal effect variants are associated with adverse reproductive outcomes more widely than represented in this study, which focuses on mothers with liveborn children with MLID. Current clinical genetic practice focuses on the proband, and there is little systematic recording of reproductive outcomes in mothers. Ascertainment of further cases, and more detailed data collection from mothers and wider families, is needed to clarify the incidence and impact of maternal effect genetic variants in reproductive and offspring health.

We previously identified five individuals within this cohort with MLID and maternal variants in *NLRP5.*^7^ At that time, *NLRP5* was the only gene to show rare variants in more than two patients. Recruitment of further patients, and advancing literature on maternal effect mutations and their developmental effects, prompted re-evaluation of our data. We have now identified 15 further families with putative maternal effect variants and offspring affected by MLID; this gives a total of 20 out of 38 pedigrees in which maternal effect variants potentially contribute to offspring MLID.

This report adds to a growing number of papers describing maternal effect variants, particularly *NLRP* gene variants, associated with offspring imprinting disturbance.^5–8^ These observations show that a proportion of MLID and atypical imprinting disorders have an underlying maternal genetic cause, particularly those where clinical problems in siblings or a history of reproductive difficulties are also present, and genetic investigation and counselling for imprinting disorders should take this into account.

10.1136/jmedgenet-2017-105190.supp3 Supplementary file 3
