# Supplementary material for: Maternal variants in NLRP and other maternal effect proteins are associated with multilocus imprinting disturbance in offspring
Source: J Med Genet. 2018 Mar 24;55(7):497–504. doi: 10.1136/jmedgenet-2017-105190 (PMC6047157; doi:10.1136/jmedgenet-2017-105190)
Supplement: Supplementary file 1 [file jmedgenet-2017-105190supp001.pdf]

Supplementary Table 1: genomic variants in pedigrees affected by multi-locus imprinting disorder

Family number	ID[1]	variant location (hg38)[2]	gene	variant info[3]	dbsnp ID[4]	ExAC_ALL[5]	ExAC_ethnicity[6]	gnomAD All[7]	SIFT[8]	PolyPhen2[9]	PROVEAN[10]	hypergeometric P-value[11]
1	BWS	Chr19:54939444 54939445:delAG	NLRP2	NM_017852.4:c.1479_1480del, p.(Arg493SerfsTer32)	rs758760659	-	-	0.0073%	-	-	-	0.016
2	SRS	Chr19:54986185:delA	NLRP2	NM_017852.4:c.2237del, p.(Asn746ThrfsTer4)	-	-	-	0.00041%	-	-	-	
3	idiopathic	Chr19:54994418-54994419:delTG	NLRP2	NM_017852.4:c.2860_2861del, p.(Cys954GlnfsTer18)	-	-	-	-	-	-	-	
4	TNDM	Chr19:54974533:C:T	NLRP2	NM_017852.4:c.314C>T, p.(Pro105Leu)	rs201724086	0.0000165	0.000015	0.0028%	T (0.15)	PD (668)	N(-2.07)	
5	SRS	Chr19:54983583:T:C	NLRP2	NM_017852.4:c.1885T>C, p.(Ser629Pro)	rs147213467	0.0009	0.0016	0.099%	D (0)	D (0.911)	D(-3.55)	
		Chr19:54990056:G:A	NLRP2	NM_017852.4:c.2401G>A, p.(Ala801Thr)	rs117066658	0.0093	0.0136	0.97%	T (0.51)	B (0.099)	T(-1.42)	
6	BWS	Chr19:54936400:C:T	NLRP7	NM_001127255.1:c.2161C>T, p.(Arg721Trp)	rs104895525	0.0001214	0.00001502	0.0057%	T (0.08)	B (0.109)	D(-4.13)	0.021
		Chr19:55445006:A:G	NLRP7	NM_001127255.1:c.2573T>C, p.(Ile858Thr)	rs776102152	-	-	0.0069%	D (0)	B (0.046)	D(-2.91)	
7	BWS/ TNDM	Chr19:54940070:A:C	NLRP7	NM_001127255.1:c.749T>G, p.(Phe250Cys)	rs78096121	0.0003954	0.0006443	0.046%	D (0)	D (0.978)	D(-7.04)	
		Chr19:54939715:A:C	NLRP7	NM_001127255.1:c.1104T>G, p.(Ile368Met)	rs1654636	0.0005806	0.0006753	0.048%	T (0.24)	B (0.06)	N(-0.91)	
8	SRS	chr19:54936405:G:A	NLRP7	NM_001127255.1:c.2156C>T, p.(Ala719Val)	rs104895526	0.001189	0.001938	0.10%	T (0.06)	PD (0.611)	D(-2.61)	
9	SRS	Chr1:17388820:G:A	PADI6	NM_207421.4:c.902G>A, p.(Arg301Gln)	rs755969432	0.0000171	0.0000308	0.0020%	D (0)	D (1)	D(-3.633)	5.00E-17
		Chr1:17394415:C:T	PADI6	NM_207421.3:c.1298C>T, p.(Pro433Leu)	rs759006424	0.0000515	0.0000774	0.0041%	D (0)	D (1)	D(-9.143)	
10	BWS	Chr1:17397091:G:A	PADI6	NM_207421.3:c.1639G>A, p.(Asp547Asn)	rs150981529	0.0007	0.0012	0.056%	T (1)	B (0.006)	N(1.738)	
		Chr1:17394024:T:C	PADI6	NM_207421.3:c.1124T>C, p.(Leu375Ser)	-	-	-	0.00041%	D (0.01)	PD (0.88)	D(-3.112)	
11	SRS	Chr1:17392197:A:G	PADI6	NM_207421.3:c.1046A>G, p.(Asp349Gly)	-	-	-	-	T (0.37)	PD (0.953)	D(-2.969)	
12	SRS		PADI6	NM_207421.3:c.433A>G, p.(Lys145Glu)	-	-	-	-	-	-	-	-
13	TNDM	Chr6:73369684:G:A	OOEP	NM_001080507.2:c.109C>T, p.(Arg37Trp)	rs189355507	0.0000166	0.0001161	0.00081%	D (0.04)	D (0.998)	D(-3.6)	0.084
14	SRS	Chr19:4930782:G:A	UHRF1	NM_013282.4:c.514G>A, p.(Val172Met)	rs753942436	0.00000837	0.0000152	0.00041%	D (0)	D (0.958)	N(-2.13)	0.12
15	BWS	Chr4:48492438:G:T	ZAR1	NM_175619.2:c.130G>T, p.(Glu44Cys)	-	-	-	-	D (0.01)	PD (0.748)	N (-2.45)	0.12

The table summarises rare genomic variants found in maternal-effect genes, in pedigrees affected by MLID. 1. the clinical presentation for which the proband was initially referred for genetic testing (BWS: Beckwith-Wiedemann syndrome; SRS: Silver-Russell syndrome; TNDM: transient neonatal diabetes mellitus. 2. the genomic location of the variant annotated in hg38/GRCh38. 3. the location of the variant in the major gene transcript (Genbank locus), exon, nucleotide change and amino-acid change. 4. SNP ID (dbSNP147). 5. minor allele frequency in ExAC (accessed June 2017). 6. minor allele frequency according to ExAC in the ethnicity of the pedigree. 7. minor allele frequency in GnomAD (accessed October 2017). 8. SIFT: D=deleterious (0-0.05); T=tolerated (0.05-1). 9. PolyPhen2: D=damaging (0.956-1); PD=possibly damaging (0.453-0.956); B=benign (0-0.453). 10. PROVEAN: D=deleterious (<-2.5); N=neutral (>-2.5). 11. Hypergeometric P-value (ExAC-all) for variants with MAF<0.001
