# Supplementary material for: Maternal variants in NLRP and other maternal effect proteins are associated with multilocus imprinting disturbance in offspring
Source: J Med Genet. 2018 Mar 24;55(7):497–504. doi: 10.1136/jmedgenet-2017-105190 (PMC6047157; doi:10.1136/jmedgenet-2017-105190)
Supplement: Supplementary file 2 [file jmedgenet-2017-105190supp002.docx]

**Supplement: Enhanced history on the families included in this study.**

Family 1 (Figure 1a): Maternal homozygous *NLRP2* c.1479_1480delAG (p.(Arg493SerfsTer32))

Family 1 of Saudi Arabian origin had two children with BWS features and hypomethylation of the KCNQ1OT1 TSS DMR; additionally MLID was present. The family reported that the two children showed a delayed development in comparison to their healthy sister. Clinically, the boy exhibited an omphalocele, neonatal hypoglycaemia and a heart defect. For his younger affected sister macroglossia was reported. In addition to these two affected children and one healthy child, the mother suffered three miscarriages. The parents of family 1 are first cousins.

Family 2: Maternal heterozygous *NLRP2* (p.(Asn746ThrfsTer4))

Proband 2 is one of non-monozygotic triplets, born after IVF treatment because of infertility. The patient exhibited a typical SRS phenotype, accounted for by hypomethylation of the H19 TSS DMR. The patient and his family have previously been described (23).

Family 3: maternal heterozygous *NLRP2* (p.(Cys954GlnfsTer18))

Proband 3 presented at the age of nearly 12 years with developmental delay, moderate to severe mental retardation, severe speech delay, microcephaly, relatively short stature and precocious puberty.

During pregnancy the mother had amniocentesis because of high maternal age, which revealed Klinefelter-Syndrome with the karyotype 47,XXY. The mother elected to continue the pregnancy. Ultrasound at that time was unremarkable. The mother and father were aged 33 and 38 years respectively at the time of his birth.

The boy was born at 27 weeks of gestation, with birth weight 465g, and occipitofrontal circumference 32cm. After birth broncho-pulmonal dysplasia was diagnosed, and artificial respiration was necessary for the first two months of life. Intracranial bleeding was excluded. Nasogastric tube feeding was necessary for the first year of life. The boy showed hypotonia, constipation, and weakness of tongue-mouth motor skills. Hypogonadism and hypospadias was diagnosed. During childhood he consistently showed growth and head circumference below the third percentile.

He has no seizures. He shows a severe speech-delay, with only a few words, though comprehension is better than active speech. His behavior is shy and he has low self-confidence. Night-sleep is good. In the night he still needs nappies. He shows accelerated bone-age and precocious puberty.

At examination aged 11 years and 7 months, his height was 139.7cm (7^th^ centile), and head circumference 49cm (-3,91 SDS). He showed facial dysmorphism, with deep set eyes, hypertelorism, short philtrum, pronounced chin, frontal and posteriorly deep set hairline, and narrow bitemporal diameter. His hands and feet were small with clinodactyly of both fifth fingers. He showed distinctive pubic and axillary hair-growth pattern, uncommon for the age of the child. Although he is not clearly overweight he shows a normal to higher weight and no dystrophy. Molecularly, hypomethylation of the H19 TSS-DMR was consistent with his features of SRS.

Proband 3 has no siblings, and his mother had no pregnancy losses. Of the mother’s two sisters, one had three pregnancy losses and one healthy child, and the other has one healthy daughter and no history of pregnancy losses.

Family 4: maternal heterozygous *NLRP2* c.314C>T (p.(Pro105Leu))

Proband 4 is the first of three children born to healthy unrelated parents; the mother additionally suffered at least two miscarriages. The mother and father were aged 19 and 22 years, respectively, at the proband’s birth. The pregnancy of the proband was complicated by polyhydramnios from 26 weeks. At birth, at 41 weeks’ gestation, his birth weight was 2955g (9^th^ centile) and his OFC 35.5cm. At 53 hours of life he presented with hyperglycaemia associated with transient neonatal diabetes mellitus (TNDM) requiring exogenous insulin; TNDM remitted within three months. By age 8 months his weight was above the 99^th^ centile and his OFC on the 50^th^ centile; aged 3 years his height and weight were above the 99^th^ centile. At this time behavioural difficulties were noted, with high activity. At age 6 he was diagnosed with an autistic spectrum disorder, with speech and language delay and specialist support in school. Diabetes recurred at age 12 years. Complete hypomethylation of PLAGL1 TSS alt-DMR accounted for his clinical features of TNDM. Of note, the younger brother of the proband was affected by anxiety, sufficiently severe to prevent school attendance, and autistic spectrum disorder. The third child was reportedly doing well in mainstream school with no problems aside from a degree of anxiety.

Family 5: Maternal heterozyous *NLRP2* c.1885T>C, (p.(Ser629Pro)) and c.2401G>A, (p.(Ala801Thr))

The proband in Family 5 is the first of two children, born when his mother and father were aged 25 and 28 years respectively. The second child is reportedly healthy, and the mother subsequently suffered one miscarriage. IUGR was detected during the first pregnancy, and the proband was delivered post-term at birth weight 1.76kg, occipitofrontal circumference (OFC) 35.5cm and birth length 43.5cm. He had bilateral radial anomalies, absent right thumb and dysmorphic left thumb. Neonatal feeding difficulties led to nasogastric tube feeding in infancy. He showed undescended testes, a single kidney, a contracture of the left elbow and bilateral fifth-finger clinodactyly, and asymmetry with the left arm and left side of the face smaller than the right. He had delayed motor milestones but no speech delay, and attended mainstream education with support for writing. He was treated with growth hormone from age 5 to 8 years. He was clinically diagnosed with SRS at age 3 years. His adult height was 153.2cm and OFC 56.3cm. Adult examination showed a high narrow palate, asymmetry of face, arms and legs with a rudimentary left thumb, and 2-3 syndactyly of the toes. The proband showed complete hypomethylation of H19 TSS DMR in blood, consistent with his clinical diagnosis of SRS.

Family 6 (Figure 1b) maternal compound heterozygous *NLRP7*: c.2161C>T (p.(Arg721Trp)); c.2573T>C (p.(Ile858Thr))

Proband 6 was a fetus ascertained by ultrasound at 19 weeks’ gestation with flat face, retrognathia, shortened humeri, omphalocele, and placental mesenchymal dysplasia. Amniocentesis revealed a normal karyotype (46,XY). Macroscopic analysis of the fetus after induced abortion confirmed the ultrasound findings, while molecular analysis in fetal lymphocytes revealed hypomethylation of KCNQ1OT1 TSS-DMR and MLID. The mother of proband 6 had two additional early pregnancy losses. The sister of mother 6 likewise suffered from recurrent pregnancy losses, but also had a healthy son. The reproductive history of the grandmother could not be ascertained.

The mother of proband 6 was compound heterozygous carrier of two variants: c.2161C>T and c.2573T>C ((p.(Arg721Trp);p.(Ile858Thr)); the latter was detectable in the fetus. Both variants have previously been reported in the literature (21,22): c.2161C>T was reported as pathogenic in families with biparental hydatidiform mole and spontaneous abortion, whereas the pathogenicity of c.2573T>C is unclear. Segregation analysis revealed that the mother and sister of mother 6 were heterozygous for c.2161C>T, but that the sister was also compound-heterozygous.

Family 7: maternal compound heterozygous *NLRP7* c.749T>G (p.(Phe250Cys)) and c.1104T>G; (p(Ile368Met))

The parents of Proband 7 had no consanguinity, no history of imprinting or congenital disorders, no fertility problems, and no ART to achieve conception. The mother and father were 26 and 33 years respectively at the proband’s birth. After a normal pregnancy, she was born at 36 weeks of gestation, with weight at 91^st^ centile and length at 99.6^th^ centile. She presented with exomphalos, repaired immediately, as well as macroglossia, feeding difficulties and transient hyperglycaemia, and was diagnosed with BWS. In childhood she displayed motor and speech delay as well as learning disabilities. At 12 years she was diagnosed with osteoporosis of the spine and hip, which was managed conservatively. Adult-onset diabetes indicated by increased weight (BMI of 25) and a perineal infection was diagnosed at 25 years, and managed with metformin and dietary changes. Further examination at this age revealed a duplex kidney system, left sided hemihypertrophy and scoliosis. She also had problematic joints and contraceptive-controlled heavy periods. She attends a college for learning independence skills and a day centre once a week.

Molecularly, MLID including both KCNQ1OT1 TSS-DMR and PLAGL1 TSS alt-DMR accounted for clinical features of both BWS and transient neonatal diabetes mellitus (TNDM). Array-CGH revealed additional material associated with the short arm of chromosome 20, which was evaluated to account for her developmental delay.

Family 8: maternal heterozygous *NLRP7* c. 2156C>T (p.(Ala719Val))

Proband 8 is the first of three children of non-consanguineous parents, with no family history of imprinting disorders or any other congenital disorders. His mother and father were aged 26 and 28 years, respectively, at his birth. After a normal pregnancy, with no reported IUGR, the patient was delivered spontaneously at 38-39 weeks of gestation, with a birthweight on the 0.4^th^ centile and reportedly normal head circumference. He presented with extra cartilage folds in the helix of the ear, a preauricular skin tag, downslanting palpebral fissures and bilateral wide sandal gap. Neonatal feeding difficulties required the use of a nasogastric tube, but glycaemic control was normal. He achieved normal motor milestones, but had mild early speech delay; but his communication and educational ability were normal, and he completed tertiary education. In review (aged 32 years) he had a facial gestalt of SRS, and right-sided hemihypertrophy, posteriorly rotated ears and fifth toe clinodactyly were noted. His measurements were: 57.6cm mean head circumference, 168.1cm height, 57.8kg weight; and other examinations were normal. Molecularly, the hypomethylation of H19 TSS-DMR in blood-derived DNA was consistent with his diagnosis of SRS.

Family 9: maternal compound heterozygous *PADI6* c.902G>A, (p.(Arg301Gln)) and c.1298C>T, (p.(Pro433Leu))

The female proband in family 9 is the only child of healthy unrelated parents, and was born when her mother and father were aged 21 and 23 years respectively. The mother reported no fertility problems or pregnancy losses. The birth weight at 38 weeks gestation was 2.1kg and birth length 44.5cm (both 2^nd^ centile), and her OFC was 33cm (25^th^-50^th^). Micrognathia was noted at birth and significant feeding problems led to NG tube feeding in the first month. Her mother reported that she showed hypoglycaemia on the neonatal ward, which was corrected by feeding. Reportedly she had early hypotonia and delay in walking. At the age of four years her height was >3SD below the mean. At four years she had low growth hormone (GH) levels, and GH was commenced at 5 years. By 8 years her height was in the 10-25^th^ centile, weight on the 90^th^ centile and OFC on 50^th^ centile. Menarche was at age 10 years (there was a family history of early menarche). At age 10 years she had mild facial asymmetry, ameliorating with age, retrognathia and crowded teeth, a hight forehead, and a broad flesh nasal tip. She had bilateral fifth finger clinodactyly and camptodactyly of all other fingers. Inability to supinate either forearm suggested radioulnar synostosis. She had no speech or language delay and was in mainstream education, but she showed fine motor delay, attributable to joint restriction.

Of note, the maternal grandfather’s family had a history of pregnancy loss; the birth of one healthy brother and one with low birthweight was followed by four stillbirths including a twin.

Proband 9 showed complete hypomethylation of both H19 TSS-DMR and MEG3 TSS-DMR, accounting for clinical features of both SRS and Temple syndrome. Two variants in *PADI6*: (p.(Arg301Gln); p(Pro433Leu)) were identified in the mother, but insufficient DNA was available from other family members to determine transmission of these variants.

Family 10: maternal *PADI6*: c.1639G>A (p.(Asp547Asn)), and c.1124T>C (p.(Leu375Ser))

The male proband of family 10 was the first child of healthy, unrelated parents (the mother aged 24 and the father 27 at his birth). After an uncomplicated pregnancy he was born at 39 weeks gestation with birth weight on the 90-97 centile, and length and head circumference above the 97^th^ centile. He presented with macroglossia and tongue asymmetry, nevus flammeus located below the nose and on the forehead, left side hemihyperplasia, ear creases and developmental delay, and slight hydrocephalus in his first year. At most recent examination, aged three years, he still manifested left-sided hyperplasia, a degree of speech difficulty, and hyperopia. There was no family history of note of imprinting or congenital disorders. The proband showed partial hypomethylation of KCNQ1OT1 TSS-DMR, accounting for his clinical diagnosis of BWS.

Family 11: maternal heterozygous *PADI6* c.1046A>G, (p.(Asp349Gly))

Proband 11 was referred for molecular SRS testing, and H19 TSS DMR hypomethylation as well as MLID were detected. He is the third child of healthy parents, the reproductive and family history in the family was unremarkable. Clinically, the patient did not show the typical features of SRS, with an NH-CSS of only 3/6 (pre- and postnatal growth restriction and asymmetry), but he was developmentally delayed.

Family 12: Maternal heterozygous *PADI6* c.433A>G, (p.(Lys145Glu))

Patient 12 is the only child of a healthy German couple. The boy was born SGA at term (weight at birth 1950g, length 45cm, OFC 34 cm). Clinical scoring resulted in a positive NH-CSS (4/6 parameters), Hypomethylation was observed for the DMRs at the IGF2R and MEG3 loci in addition to the diease-specific H19 TSS DMR.

Family 13: maternal homozygous *OOEP* (c.109C>T, p.(Arg37Trp))

The proband is the third child of healthy unrelated parents. His elder brother and sister are 15 and 12 years older, respectively. The child had birth weight 2100g at 40wg. He presented with transient neonatal diabetes mellitus at 28 days, which remitted at 3.5 months; he also showed a small patent ductus arteriosus, and left sided pelvic renal dilatation. The child is reportedly healthy at present, though with unspecified developmental delay.

Proband 13 showed complete hypomethylation of PLAGL1 TSS alt-DMR, accounting for his clinical diagnosis of TNDM; and in the context of MLID also showed partial hypomethylation at SNURF TSS-DMR. Of note, the missense variant in *OOEP*, predicted to be deleterious, was present homozygously in the mother, and heterozygously in the proband and father.

Family 14: maternal heterozygous *UHRF1* c.514G>A (p.(Val172Met))

Proband 14 is one of discordant monozygotic (monochorionic, diamniotic) twins; her co-twin is reportedly clinically and epigenetically normal, though she shared the variant in *UHRF1*. The twins were born when their mother and father were 32 and 25 years, respectively. At the time of their conception, the mother was taking oral contraceptives. The pregnancy was complicated by vaginal bleeding at 12 weeks gestation, and then by pre-eclampsia before onset of spontaneous labour at 31 weeks. The birth weight of the affected and unaffected twins were 1.549kg and 1.86kg respectively, and the proband’s OFC was 27.9cm. Neonatal features of note included an inguinal hernia requiring surgical correction, no abnormality of glycaemia, and a glabellar naevus flammeus. Neonatal ultrasound showed hepatomegaly and small kidneys, which were thought to be possibly asymmetric. Neonatal kidney failure was managed conservatively and resolved at 1 month. After discharge from hospital at 5 weeks, the proband suffered a cyanotic episode at 8 weeks, and thereafter repeatedly had low oxygen levels; she was monitored and treated with oxygen for 9 months.

At around 3 years, ultrasound showed renal dysplasia (abnormal echogenicity and impaired corticomedullary differentiation) and abnormal kidney function; and further ultrasound at 5.5 years showed the same features and additionally small simple cysts, consistent with bilateral renal aplasia.

A diagnosis of SRS was first considered at eight months. Clinical genetic assessment at this time noted hemihypotrophy of the right arm and leg, and also frontal bossing and epicanthic folds, although no triangular facies or fifth-finger clinodactyly characteristic of SRS were noted. She showed slight developmental delay, reaching milestones 6-8 months behind her twin; she walked at 21m and spoke her first words at 2 years of age. She had strabismus, and moderate hearing loss at moderate/high frequencies.

On examination at 6.5y, her height was 110.8cm and weight 17.2kg (both 3^rd^ centile). She had right-sided hemihypotrophy of the arm and leg, fifth-finger clinodactyly, wide-spaced teeth, normal tongue, mouth and ears, no café-au-lait spots, normal abdominal wall, and mild frontal bossing. Her twin’s height and weight were 123cm and 23.8kg, both 50-75^th^ centile.

Proband 14 showed partial hypomethylation of H19 TSS DMR, accounting for her clinical diagnosis of SRS; notably, she also showed partial hypomethylation of KCNQ1OT1 TSS-DMR. Of note, the *UHRF1* variant *UHRF1* variant (c.514G>A, p.(Val172Met)) was present in the mother and both twins.

Family 15: maternal heterozygous *ZAR1* c.170G>T, (p.(Glu44Cys)).

The proband in Family 15 is the third of three children; between the first and second children the mother suffered one miscarriage. At the proband’s birth, the mother and father were aged 36 and 37 years, respectively. The birth weight of the patient was 4.44kg, above the 98th centile. She was macrosomic; at age 1 year, her BMI was 23, markedly above the 99.6th centile, and she was referred for endocrine investigation. At this time, she was noted to have mild macroglossia, but no other feature of BWS. Genetic testing for BWS was inconclusive at that time. After some lessening of macrosomia between ages 3 and 7, during which she received some speech therapy, more extensive testing for imprinting disorders was undertaken aged 11 years. This revealed BWS-MLID, with mild hypomethylation of either KCNQ1OT1 TSS-DMR or GNAS-AS1 TSS DMR potentially accounting for her accelerated growth. Aged 12 years her BMI remains above the 98th percentile.
