# Supplementary material for: Maternal variants in NLRP and other maternal effect proteins are associated with multilocus imprinting disturbance in offspring
Source: J Med Genet. 2018 Mar 24;55(7):497–504. doi: 10.1136/jmedgenet-2017-105190 (PMC6047157; doi:10.1136/jmedgenet-2017-105190)
Supplement: Supplementary file 3 [file jmedgenet-2017-105190supp003.pdf]

Supplementary Table 2: Methylation disturbance at imprinted loci in probands with multi-locus imprinting disorder

proband number	gene	DIRAS3 TSS DMR	PLAGL1 TSS alt-DMR	IGF2R Int2 DMR	GRB10 alt-TSS DMR	MEST alt-TSS DMR	H19 TSS DMR	KCNQ1OT1 TSS DMR	MEG3 TSS DMR	SNURF TSS DMR	IGF1R Int2 DMR	PEG3 TSS DMR	GNAS-AS1 TSS DMR	GNAS A/B TSS DMR
1	*NLRP2*	ND	**0.11**	ND	**0.103**	**0.13**	0.5	**0.12**	0.5	0.5	ND	0.5	0.5	0.5
2	*NLRP2*	ND	0.5	**0.07**	0.5	**0**	**0.19**	**0.06**	0.5	0.5	ND	ND	0.5	0.5
3	*NLRP2*	ND	0.5	ND	**0.09**	**0.17**	**0.16**	**0.18**	**0.1**	0.5	ND	0.5	**0.21**	**0.14**
4	*NLRP2*	**0.25**	**0**	**0.25**	0.5	**0**	0.5	0.5	0.5	0.5	**0.25**	0.5	0.5	0.5
5	*NLRP2*	0.5	0.5	**0**	0.5	0.5	**0**	0.5	0.5	0.5	0.5	0.5	0.5	0.5
6	*NLRP7*	ND	0.5	**0.42**	**0.23**	**0.1**	0.5	**0.06**	0.5	0.5	ND	0.5	0.5	**0.21**
7	*NLRP7*	0	**0**	**0**	0.5	**0**	0.5	**0**	0.5	0.5	**0.09**	0.5	0.5	0.5
8	*NLRP7*	0.5	0.5	**0.13**	0.5	0.5	**0.14**	0.5	0.5	0.5	**0.05**	0.5	0.5	0.5
9	*PADI6*	ND	0.5	0.5	0.5	0.5	**0**	0.5	**0**	**0.33**	**0.33**	0.5	0.5	**0.07**
10	*PADI6*	0.5	0.5	**0.2**	**0**	**0.25**	**0.33**	**0.25**	0.5	0.5	**0.25**	0.5	0.5	0.5
11	*PADI6*	ND	0.5	**0.36**	**0.25**	**0.25**	**0.43**	0.5	**0.37**	**0.33**	ND	0.5	**0.27**	**0.24**
12	*PADI6*	ND	0.5	**0.18**	0.5	0.5	**0.35**	0.5	**0.38**	0.5	ND	ND	0.5	0.5
13	*OOEP*	**0.1**	**0**	**0**	**0.2**	0.5	0.5	0.5	ND	**0.14**	**0.25**	0.5	0.5	0.5
14	*UHRF1*	0.5	**0.33**	**0.33**	0.5	0.5	**0.33**	**0.33**	0.5	0.5	**0.2**	**0.33**	**0.33**	0.5
15	*ZAR1*	**0.42**	0.5	0.5	0.5	0.5	0.5	**0.42**	0.5	0.5	**0.34**	0.5	**0.42**	**0.34**

DNA methylation at selected genomic imprints (indicated by row headers) was measured by targeted testing in MLID patients (indicated in leftmost column). Figures state the proportional DNA methylation for each patient at each locus: for example, a methylation index of 0.5 is equivalent to normal hemimethylated status; methylation index of 0 indicates no detectable DNA methylation. ND:not done In Probands 1, 9 and 11 DNA methylation was also assessed by Illumina HumanMethylation 450K array (Refs 3, 48).
